# Screening and validating genes associated with cuproptosis in systemic lupus erythematosus by expression profiling combined with machine learning

**DOI:** 10.17305/bb.2024.10996

**Published:** 2024-10-02

**Authors:** Zhongbin Xia, Ruoying Cheng, Qi Liu, Yuxin Zu, Shilu Liao

**Affiliations:** 1 Health Management Medicine Department, The Second Affiliated Hospital, Jiangxi Medical College, Nanchang University, Nanchang, China; 2 The Second Affiliated Hospital, Jiangxi Medical College, Nanchang University, Nanchang, China

**Keywords:** Systemic lupus erythematosus, machine learning, cuproptosis, single-cell RNA-sequencing

## Abstract

Cell death has long been a focal point in life sciences research, and recently, scientists have discovered a novel form of cell death induced by copper, termed cuproptosis. This paper aimed to identify genes associated with cuproptosis in systemic lupus erythematosus (SLE) through machine learning, combined with single-cell RNA sequencing (scRNA-seq), to screen and validate related genes. The analytical results were then experimentally verified. Two published microarray gene expression datasets (GSE65391 and GSE61635) from SLE and control peripheral blood samples were downloaded from the GEO database. The GSE65391 dataset was used as the training group, while the GSE61635 dataset served as the validation group. Differentially expressed genes from GSE65391 identified 12 differential genes. Nine diagnostic genes, considered potential biomarkers, were selected using the least absolute shrinkage and selection operator and support vector machine recursive feature elimination analysis. The receiver operating characteristic (ROC) curves for both the training and validation groups were used to calculate the area under the curve to assess discriminatory properties. CIBERSORT was used to assess the relationship between these diagnostic genes and a reference set of infiltrating immune cells. scRNA-seq data (GSE162577) from SLE patients were also obtained from the GEO database and analyzed. Experimental validation of the most important SLE biomarkers was performed. Twelve significantly different cuproptosis-related genes were identified in the GSE65391 training set. Immune cell analysis revealed 12 immune cell types and identified nine signature genes, including *PDHB*, glutaminase (*GLS)*, *DLAT*, *LIAS*, *MTF1*, *DLST*, *DLD*, *LIPT1*, and *FDX1*. In the GSE61635 validation set, seven genes were weakly expressed, and two genes were strongly expressed in the treatment group. According to the ROC curves, *PDHB* and *GLS* demonstrated significant diagnostic value. Additionally, correlation analysis was conducted on the nine characteristic genes in relation to immune infiltration. The distribution of key genes in immune cells was determined using scRNA-seq data. Finally, the mRNA expression of the nine diagnostic genes was validated using qPCR.

## Introduction

Systemic lupus erythematosus (SLE) is characterized by inflammation and autoimmune tissue destruction affecting multiple organs, occurring more frequently in young women [1, 2]. Extensive research strongly supports the notion that dysregulation of cell death pathways and defective clearance of dead material trigger autoimmunity, promoting the onset and progression of SLE [3]. The inflammatory cell death pathway is closely linked to SLE pathogenesis, and inhibiting this process while enhancing the clearance of dead material at various stages may offer a promising therapeutic strategy for SLE treatment [4–6]. In August 2021, researchers identified the key role of neutrophil ferroptosis in autoimmune diseases in both humans and mice, providing insights into the regulatory mechanisms that drive specific forms of neutrophil death in SLE patients [7]. Understanding the pathogenesis of SLE is crucial for accurate diagnosis, reasoning, and treatment [7].

Cell death is a natural phenomenon and has been a major focus in life sciences research. Over the past few decades, various forms of cell death have been identified, each relying on distinct proteins to activate and execute its specific pathway. The mechanisms of cell death differ, with common forms, including apoptosis, pyroptosis, necrosis, and ferroptosis [8–11]. Among these, ferroptosis, first identified in 2012, has garnered significant attention, as it precedes the discovery of cuproptosis [7, 12–14]. Copper, like iron, is found in all living organisms and is an essential micronutrient for normal biological functions. It is tightly regulated at low levels in mammalian cells, but when copper ion concentrations exceed the threshold for homeostasis, they become cytotoxic [12, 15, 16]. In 2022, researchers introduced the term “cuproptosis” to describe a controlled form of cell death, distinct from apoptosis, necrosis, and ferroptosis. Cuproptosis primarily occurs through the accumulation of intracellular copper ions. These ions have been shown to interact significantly with the tricarboxylic acid (TCA) cycle. When copper ions accumulate beyond a critical concentration, they bind directly to lipid-acylated components of the TCA cycle, causing protein aggregation and imbalance, which in turn disrupts the TCA process, triggering toxic stress on proteins and leading to cell death [15].

With the discovery of cuproptosis, numerous bioinformatic analyses have explored its relationship with immune responses and disease. In November 2022, Yuan et al. [17] performed a differential expression analysis combined with machine learning, discovering that cuproptosis may contribute to the progression of Crohn’s disease by inducing immune responses and metabolic dysfunction. In July 2022, Lai et al. [18] utilized WGCNA and other analyses to explore the relationship between cuproptosis and Alzheimer’s disease, developing a predictive model to assess pathological outcomes in Alzheimer’s patients. In March 2022, Chen et al. [19], using the dataset from ssGSEA, receiver operating characteristic (ROC) curves, and Autodock Vina, found that cuproptosis-related pathway regulation could significantly influence the development and progression of inflammatory bowel disease. Given the connection between cell death and the pathogenesis of SLE, it is reasonable to infer an undiscovered relationship between SLE and cuproptosis [7]. Cuproptosis is a copper-dependent programmed form of cell death, distinct from apoptosis, pyroptosis, necrosis, and autophagy. In cuproptosis, copper directly binds to lipoproteins in the TCA cycle, leading to lipoprotein aggregation and loss of iron–sulfur clusters, inducing protein toxicity stress, and ultimately cell death. However, its role in SLE remains unclear, and understanding cuproptosis could shed light on the mechanisms underlying SLE.

Recently, researchers have suggested that advances in machine learning applications could aid in predicting changes in disease activity in SLE [20]. In addition, the application of single-cell RNA sequencing (scRNA-seq) in recent years has deepened our understanding of RNA transcription heterogeneity and complexity within individual cells [21]. By integrating various expression profiling techniques, including microarrays, researchers have uncovered the molecular characteristics of immune microenvironments and the heterogeneity of immune cells across multiple diseases [22–24]. This technology holds great potential for transforming current disease diagnosis and treatment protocols [21]. In this study, we employ machine learning combined with single-cell sequencing and animal experiments to identify, screen, and validate cuproptosis-related mechanisms in SLE, providing new insights into SLE pathogenesis and treatment.

## Materials and methods

### Microarray data information

Gene expression data from the GSE65391 and GSE61635 datasets were downloaded from the NCBI GEO database. Single-cell sequencing data were derived from GSE162577. The GSE65391 dataset included 924 patients with SLE and 72 controls, based on the GPL10558 platform. The GSE61635 dataset included 79 patients with SLE and 30 controls, collected from blood samples, and was based on the GPL570 platform. In total, 1003 SLE patients and 102 healthy controls were analyzed. The GSE65391 dataset was used as the training group, while the GSE61635 dataset served as the validation group. The SLE patients were classified as the treatment group, and the healthy controls as the control group. Probes representing multiple genes were removed and replaced with gene symbols according to probe annotation files. The mean value of the probes was calculated as the final gene value. The data were analyzed using R version 4.2.2. The datasets GSE65391 and GSE61635 were chosen for their extensive coverage of gene expression profiles in SLE patients, providing a solid basis for identifying cuproptosis-related genes.

### Identification and visualization of differentially expressed genes (DEGs)

The “limma” package in R (available at http://www.bioconductor.org/limma/) was used for data preprocessing, including background correction, normalization, and differential gene expression analysis between GSE65391 and the 18 cuproptosis-associated genes. After adjusting for the false discovery rate (*P* value < 0.05) and the threshold points for |log FC| > 1.0 as DEGs, 12 differential genes were identified. The “pheatmap” and “ggpubr” R packages were used to create heatmaps and boxplots for visualizing the differential genes. Cuproptosis-related genes were identified from the GeneCards database, and their expression in SLE patients and healthy controls was analyzed using the training set GSE65391. Since SLE presents with prominent skin lesions, genes related to the skin lesion-associated cuproptosis pathway were downloaded and intersected with the SLE DEGs, leading to the identification of 12 key genes.

### Immune cell infiltration analysis by CIBERSORT

The “RCircos” package in R was used to visualize the chromosomal locations of the 18 cuproptosis-associated genes. Additionally, immune cell infiltration was calculated using the bioinformatics algorithm CIBERSORT (https://cibersortx.stanford.edu/), referencing 22 immune cell types to estimate immune cell abundance. The “corrplot” R package was employed to compute and display correlation coefficients between the 12 differential genes.

### Screening for the best diagnostic biomarkers for SLE

To identify important prognostic markers, we employed two machine learning algorithms. First, we used the “glmnet” package in R for the least absolute shrinkage and selection operator (LASSO) regression algorithm to screen 11 characteristic genes for SLE from the 12 differential genes. The support vector machine (SVM), a supervised machine learning algorithm, was then applied for regression and classification [25]. To identify the optimal diagnostic gene biomarkers for SLE, both the LASSO and SVM recursive feature elimination (SVM-RFE) were used to screen the characteristic genes. The “e1071” package in R was used to build the SVM model. The SVM-RFE method removes the least important features and selects the most important ones based on classifier weights [26]. After applying SVM-RFE, ten characteristic genes for SLE were identified from the 12 differential genes. We then used the “venn” package in R to identify nine intersecting genes between those screened by LASSO and SVM-RFE. These nine intersecting genes were selected as diagnostic markers for SLE. Additionally, we used the “ggpubr” and “limma” packages in R to perform differential analysis on these nine genes in the GSE61635 dataset (used as the validation group) to observe their differences between control and treatment groups. Statistical analysis was performed using R software v4.0.3 (^*^P < 0.05, ^**^P < 0.01, ^***^P < 0.001).

### Diagnostic value of feature biomarkers in SLE

To evaluate the accuracy of the nine diagnostic genes, we constructed ROC curves for both the validation and training groups. The area under the curve (AUC) values for the nine genes were calculated by numerically integrating the ROC curves. The corresponding cutoff point for the ROC curve was determined using Youden’s index. A binary regression model was then used to calculate sensitivity, specificity, and 95% confidence intervals (CIs).

### Correlation analysis of diagnostic genes with immune cells

Correlation analysis and scatter plots were generated for the nine diagnostic genes and 22 reference sets of immune cells. The Spearman method, implemented using the “Hmisc” R package, was used to analyze the correlation between the expression levels of the nine diagnostic genes and the 22 immune cell reference sets. The results were visualized using graphical techniques from the “lollipop” package. A *P* value of <0.05 was considered to indicate a significant correlation.

### Processing and analysis of scRNA-seq data

The scRNA-seq data (GSE162577) of peripheral blood mononuclear cells (PBMCs) from SLE patients were downloaded from the GEO database (https://www.ncbi.nlm.nih.gov/geo/). Seurat (version 4.0.5) was used for cell quality control and analysis of the scRNA-seq data. The data were processed in R using the dplyr, future, and DoubletFinder packages. After quality control and normalization of the scRNA-seq data from three samples, cells with at least 300 and no more than 4000 genes, and a mitochondrial gene percentage of less than 10%, were selected. Low-quality cells were filtered, and violin plots were generated using the following criteria: (nFeature_RNA > 200, nFeature_RNA < 7000, and percent.mt < 20%). The data were dimensionally reduced, and 10k genes were screened, followed by normalized linear regression to obtain a principal component analysis (PCA) map. Marker genes were identified, and cell types were annotated after clustering, resulting in uniform manifold approximation and projection (UMAP) visualization. The t-distributed stochastic neighbor embedding (t-SNE) algorithm was also used to reduce the nonlinear dimensionality of the scRNA-seq data [27].

Finally, violin plots and bubble plots were generated to analyze the expression levels of *PDHB* and glutaminase (*GLS)* in the main cell types. Seurat (version 4.0.5) was used for cell quality control and analysis of scRNA-seq data. To visualize high-dimensional scRNA-seq data, we applied two widely used dimensionality reduction techniques: UMAP and t-SNE. These methods allowed us to reduce the data to two dimensions, facilitating the visualization of cellular heterogeneity. Both algorithms are non-linear dimensionality reduction methods that help preserve local and global data structures, aiding in the identification of distinct cell populations in SLE samples. Importantly, these visualizations do not affect downstream analysis, such as gene expression profiling or cell clustering, but enhance the interpretability of the results.

### Animals

Thirty female 55-day-old MRL/lymphocyte proliferation (lpr) SLE mice were purchased from Cyagen Research Centre for Model Organisms, Cyagen Biosciences (Jiangsu, China). Thirty female -week-old C57BL/6 mice were purchased from GemPharmatech Co., Ltd. (Jiangsu, China). MRL/lpr mice are produced by introducing an lpr mutation in MRL mice, which leads to an abnormal increase in lymphocytes, resulting in many pathological features similar to human SLE, including the production of autoantibodies, multiple organ inflammatory lesions, and lupus nephropathy. Therefore, MRL/lpr mice are considered an ideal model for studying the pathological mechanisms and potential treatments of SLE. All the mice were euthanized using CO_2_ inhalation, with death occurring between 30 s and 30 min. Next, we verified the diagnostic value of *GLS* and *PDHB* by obtaining blood samples from the mice. MRL/lpr SLE mice were used as the SLE group, while C57BL/6 mice served as the healthy control group. Both MRL/lpr and C57BL/6 mice were maintained under specific pathogen-free conditions at the Experimental Animal Center of The Second Affiliated Hospital of Nanchang University. The study protocol was approved by the Ethics Committee of The Second Affiliated Hospital of Nanchang University. All experiments were conducted in compliance with ethical standards, ensuring humane treatment of animals, and conforming to international guidelines and human moral and ethical standards.

### Total RNA extraction and RT-qPCR

To detect the mRNA expression of *GLS* and *PDHB* in the blood, total RNA was extracted from the model using RNA Trizol reagent (Invitrogen, Carlsbad, CA, USA). GAPDH was used as the reference gene for qPCR normalization. According to the manufacturer’s instructions, cDNA synthesis was carried out using a reverse transcription kit (Guangzhou Ribobio Co., Ltd.). The qRT-PCR analysis was performed using the LightCycler 480 Real-Time PCR System. Related lncRNA expression levels were calculated using the 2^−ΔΔCT^ method. The sequences were as follows: *GLS* primers: 5′-GGCAGTTTGCGTTCCATGTTG-3′ (forward) and 5′-GCGGCAAACAGAAGGTTTATCA-3′ (reverse); *PDHB* primers: 5′-AAGGCAAGGGACCCACATC-3′ (forward) and 5′-CGTAAGGCATAGGGACATCAGC-3′ (reverse).

### Ethical statement

This research was approved by the Medical Ethics Committee of The Second Affiliated Hospital of Nanchang University. Sample acquisition and usage were performed in accordance with the approved guidelines. All methods adhered to relevant regulations and were reported in accordance with the ARRIVE guidelines (https://arriveguidelines.org) for the reporting of animal experiments.

### Statistical analysis

All data analysis was performed using R, and graphs were generated with GraphPad Prism 8 software (GraphPad Software, CA, USA). Statistical analyses were conducted in RStudio (version 4.3). The LASSO regression algorithm was utilized to identify genes significantly associated with the differentiation between SLE and control groups, using the “glmnet” package in R. ROC curves and AUC were used to determine the diagnostic efficacy of biomarkers. The relationship between gene biomarker expression and infiltrating immune cells was analyzed using Spearman’s correlation. Data were analyzed using *t*-tests. If the *P* value was less than 0.05, the null hypothesis was rejected, indicating statistically significant results.

## Results

### Differential expression of cuproptosis-related genes in SLE

**Figure 1.**
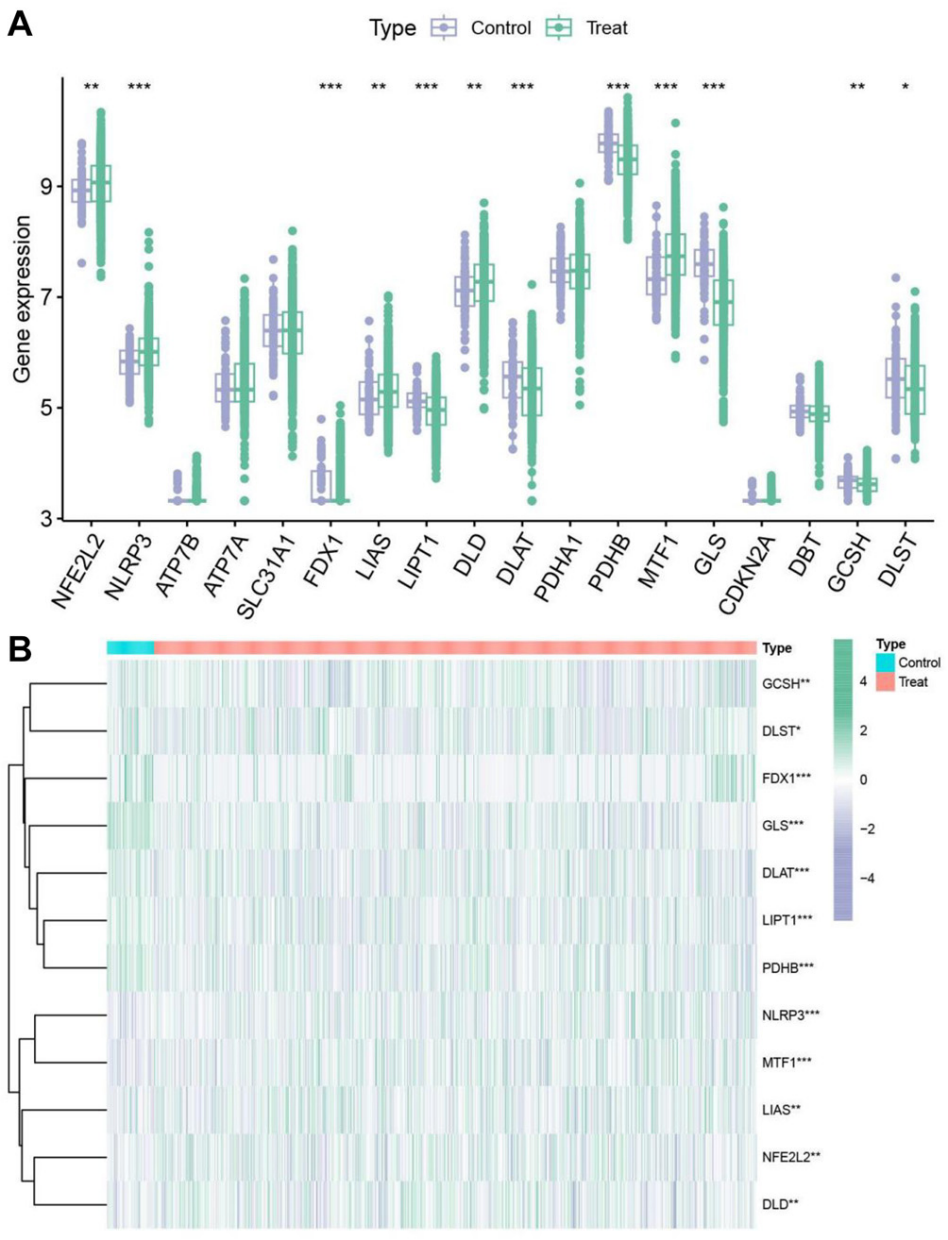
**Differential expression of cuproptosis-related genes in SLE.** (A) Analysis of the expression of 18 cuproptosis-related genes in SLE patients and healthy controls. Genes with high expression in the disease group are shown in red, while genes with low expression are shown in blue. *P < 0.05; **P < 0.01. (B) Heatmap showing the expression patterns of 12 cuproptosis-related genes in the control and treatment groups. The color gradient represents the level of gene expression, with red indicating higher expression and blue indicating lower expression levels. SLE: Systemic lupus erythematosus.

The results indicated that *NFE2L2*, *NLRP3*, *DLAT*, *LIAS*, and *MTF1* were highly expressed in SLE patients, and *LIPT1*, *DLAT*, *PDHB*, *GLS*, *GCSH*, *LIAS*, and *FDX1* were significantly downregulated in SLE patients (Figure 1A). The heatmap visualizes the differences in the expression of these 12 genes between the control and treatment groups (Figure 1B).

### Correlation analysis of cuproptosis DEGs

**Figure 2.**
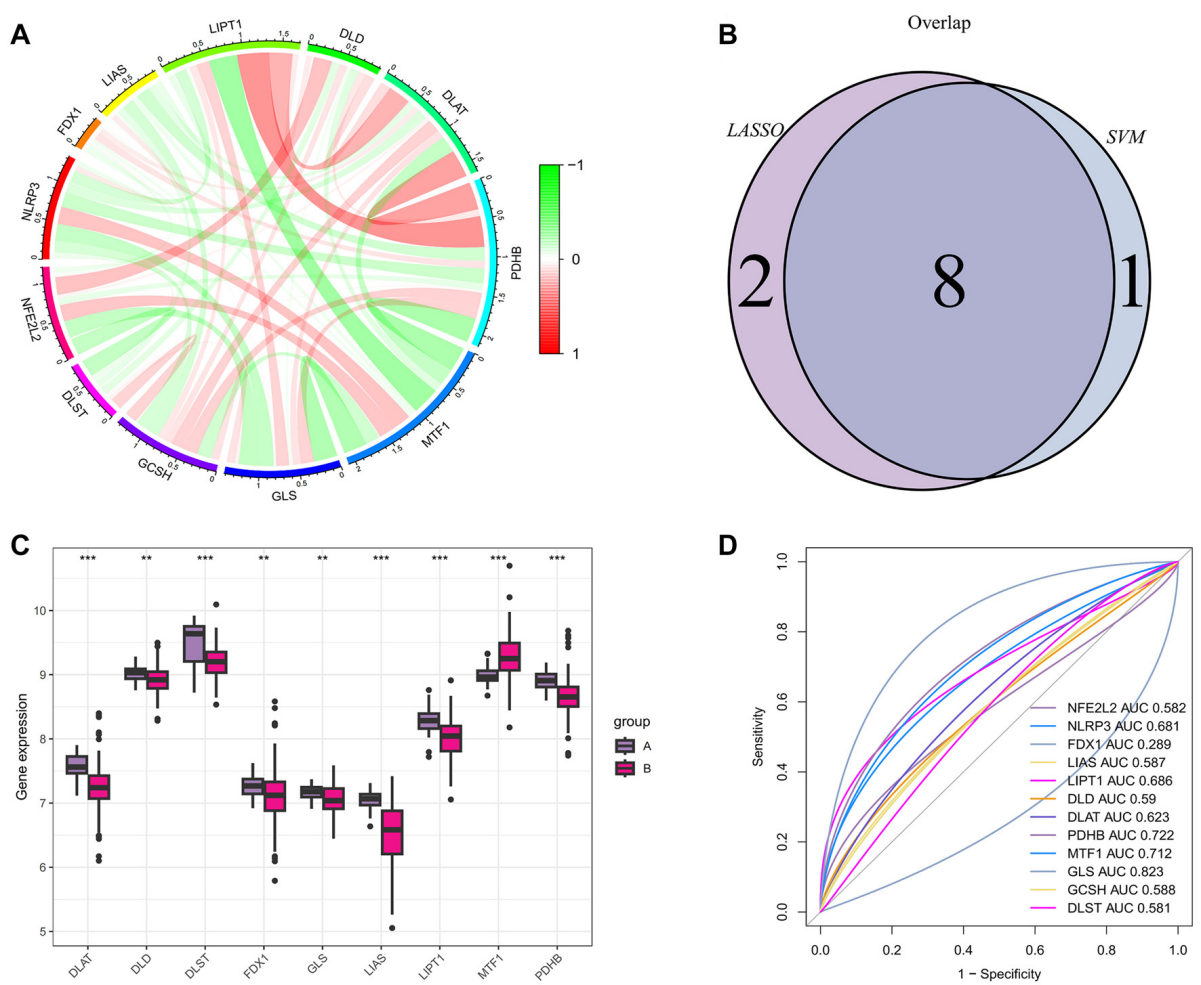
**Correlation analysis of cuproptosis DEGs with screening and validation of cuproptosis-related signatures.** (A) Circos plot of 12 DEGs; (B) Nine genes were selected by the algorithm; (C) Box and whisker plot of nine genes between the treatment and control groups using the ggpubr and limma packages of R; (D) The corresponding AUC of nine genes. DEG: Differentially expressed gene; AUC: Area under the curve.

We also examined the correlation between these genes, illustrating their relationships via the size ratio of each arc in the circos diagrams (Figure 2A). *GLS* and *MTF1*, *LIPT1* and *MTF1*, and *PDHB* and *MTF1* showed significant negative correlations. *DLAT* and *LIPT1*, *LIAS* and *PDHB*, and *PDHB* and *LIPT1* showed significant positive correlations.

### Screening and validation of cuproptosis signature genes

We used the Lasso regression algorithm and the SVM-RFE algorithm to select the most important signature genes associated with the cuproptosis pathway in SLE. The Lasso regression algorithm identified 11 significantly different signature genes. By retaining the validation set from the SVM-RFE algorithm, we obtained ten signature genes. Using Venn diagrams, we identified nine overlapping genes between the LASSO and SVM-RFE approaches as cuproptosis signature genes (Figure 2B). We further analyzed the expression differences of these nine signature genes between the control and treatment groups in the validation set (GSE61635) (Figure 2C). The expression levels of *LIPT1*, *PDHB*, *DLAT*, *DLD*, *FDX1*, and *LIAS* were higher in the control group compared to the treatment group, while *MTF1* and *GLS* were significantly lower in the control group compared to the treatment group.

### Predicting the diagnostic value of signature genes in SLE

ROC curves were used to assess the diagnostic potential of these nine signature genes for cuproptosis in SLE (Figure 2D). Predictions were performed on both the training and validation sets. The results indicated that *PDHB*, *MTF1*, *DLAT*, *LIAS*, and *DLAT* had good diagnostic values in the training group, while *PDHB*, *GLS*, and *MTF1* showed good AUC values in the validation group. The AUC values for the control and treatment groups are summarized in Table 1, where significant differences in the expression of these signature genes between the treatment and control groups were observed.

**Table 1:** ROC curves for the nine signature genes in the control group

**Gene**	**AUC**	**95% CI**
*PDHB*	0.798	0.713–0.877
*GLS*	0.542	0.430–0.620
*LIPT1*	0.687	0.581–0.784
*MTF1*	0.826	0.743–0.897
*DLAT*	0.768	0.682–0.848
*DLD*	0.617	0.519–0.713
*DLST*	0.747	0.624–0.859
*FDX1*	0.561	0.457–0.666
*LIAS*	0.836	0.761–0.901

ROC: Receiver operating characteristic; AUC: Area under the curve; CI: Confidence interval; GLS: Glutaminase.

### Analysis of the correlation between two signature genes in immune infiltration cell

**Figure 3.**
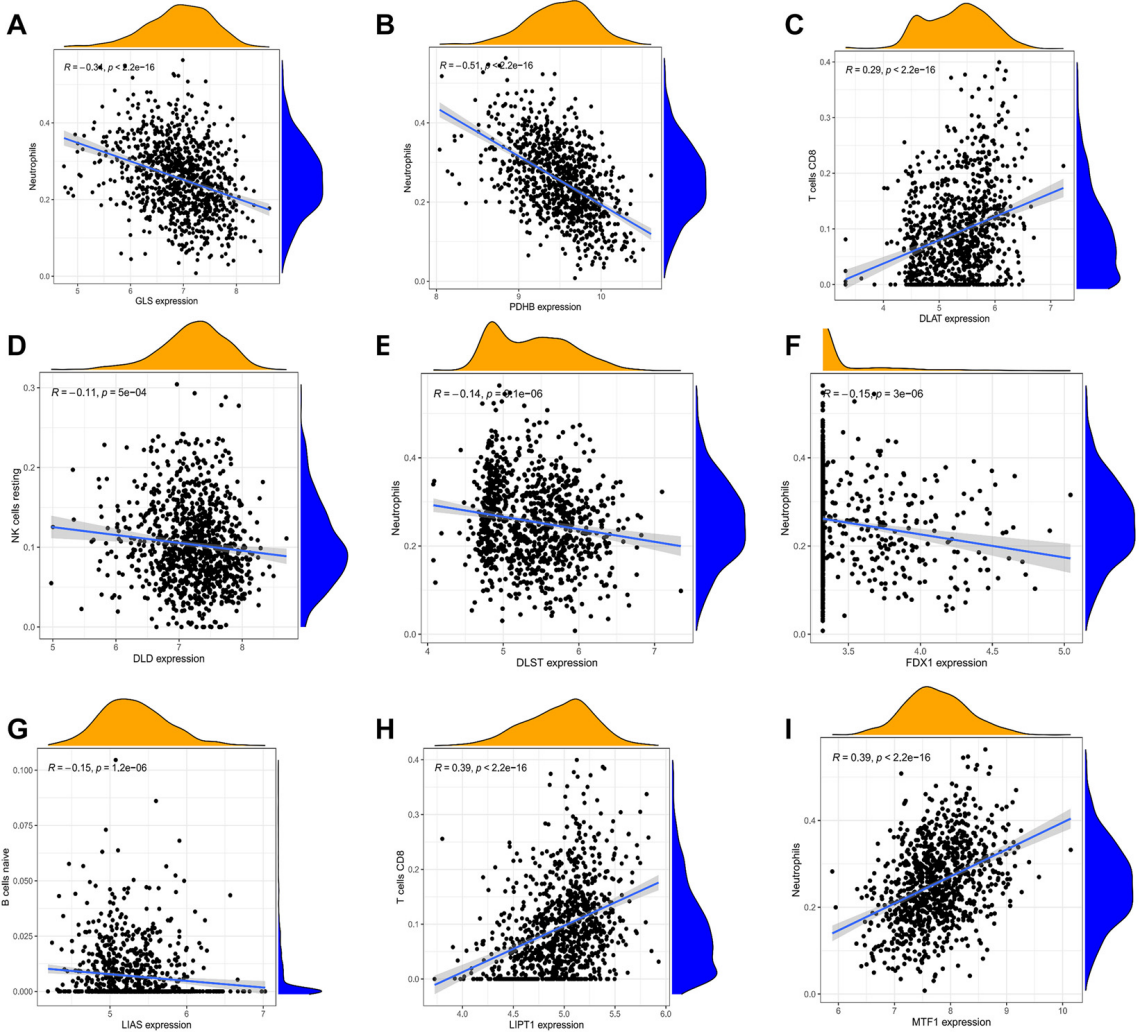
**The immune-correlation analyses between the expression of individual genes.** (A) Correlation between *GLS* and infiltrating immune cells in SLE patients using Spearman’s rank correlation analysis. The *R* value indicates correlation, and *R* value > 0 indicates a positive correlation; *P* value indicates significance. (B) Correlation between *PDHB* and infiltrating immune cells in SLE patients using Spearman’s rank correlation analysis. The *R* value indicates correlation, and *R* value > 0 indicates a positive correlation; *P* value indicates significance. (C) Correlation between *DLAT* and infiltrating immune cells in SLE patients using Spearman’s rank correlation analysis. The *R* value indicates correlation, and *R* value > 0 indicates a positive correlation; *P* value indicates significance. (D) Correlation between *DLD* and infiltrating immune cells in SLE patients using Spearman’s rank correlation analysis. The *R* value indicates correlation, and *R* value > 0 indicates a positive correlation; *P* value indicates significance. (E) Correlation between *DLST* and infiltrating immune cells in SLE patients using Spearman’s rank correlation analysis. The *R* value indicates correlation, and *R* value > 0 indicates a positive correlation; *P* value indicates significance. (F) Correlation between *FDX1* and infiltrating immune cells in SLE patients using Spearman’s rank correlation analysis. The *R* value indicates correlation, and *R* value > 0 indicates a positive correlation; *P* value indicates significance. (G) Correlation between *LIAS* and infiltrating immune cells in SLE patients using Spearman’s rank correlation analysis. The *R* value indicates correlation, and *R* value > 0 indicates a positive correlation; *P* value indicates significance. (H) Correlation between *LIPT1* and infiltrating immune cells in SLE patients using Spearman’s rank correlation analysis. The *R* value indicates correlation, and *R* value > 0 indicates a positive correlation; *P* value indicates significance. (I) Correlation between *MTF1* and infiltrating immune cells in SLE patients using Spearman’s rank correlation analysis. The *R* value indicates correlation, and *R* value > 0 indicates a positive correlation; *P* value indicates significance. SLE: Systemic lupus erythematosus; GLS: Glutaminase.

We performed a correlation analysis between the expression of nine diagnostic genes and immune cell populations (Figure 3). The results are presented in Table 2.

**Table 2:** Correlation analysis between the expression of the nine signature genes and immune cells

**Gene**	**Positive correlation**		**Negative correlation**	
	**Immune cells**	**Value**	**Immune cells**	**Value**
*PDHB*	B cells naive	*R* ═ 0.35, *P* < 2.2e-16	B cells naive B cells memory	*R* ═ −0.2, *P* ═ 1.4e-10
	NK cells resting	*R* ═ 0.14, *P* ═ 9.9e-06	Dendritic cells activated	*R* ═ −0.2, *P* ═ 7e-11
	T cells CD4 memory activated	*R* ═ 0.067, *P* ═ 0.034	Macrophages M0	*R* ═ −0.29, *P* < 2.2e-16
	Plasma cells	*R* ═ 0.084, *P* ═ 0.0082	Mast cells activated	*R* ═ −0.14, *P* ═ 1.8e-05
	Dendritic cells resting	*R* ═ 0.067, *P* ═ 0.033	Mast cells resting	*R* ═ −0.079, *P* ═ 0.012
	T cells CD4 memory resting	*R* ═ 0.23, *P* ═ 4.4e-13	Monocytes	*R* ═ −0.064, *P* ═ 0.044
	T cells CD8	*R* ═ 0.51, *P* < 2.2e-16	Neutrophils	*R* ═ −0.51, *P* < 2.2e-16
	T cells regulatory (Tregs)	*R* ═ 0.088, *P* ═ 0.0052	T cells gamma delta	*R* ═ −0.16, *P* ═ 2.1e-07
*GLS*	B cells naive	*R* ═ 0.23, *P* ═ 8.8e-14	NK cells activated	*R* ═ −0.089, *P* ═ 0.0051
	NK cells resting	*R* ═ 0.12, *P* ═ 9e-05	Monocytes	*R* ═ −0.14, *P* ═ 1.6e-05
	T cells CD4 memory resting	*R* ═ 0.29, *P* < 2.2e-16	Dendritic cells activated	*R* ═ −0.18, *P* ═ 6.2e-09
	T cells CD4 naive	*R* ═ 0.086, *P* ═ 0.0067	Macrophages M0	*R* ═ −0.2, *P* ═ 4.1e-10
	T cells CD8	*R* ═ 0.26, *P* < 2.2e-16	Mast cells resting	*R* ═ −0.24, *P* ═ 5.2e-15
			B cells memory	*R* ═ −0.093, *P* ═ 0.0031
			Neutrophils	*R* ═ −0.34, *P* < 2.2e-16
*DLAT*	T cells CD8	*R* ═ 0.29, *P* < 2.2e-16	T cells CD4 naive	*R* ═ −0.071, *P* ═ 0.025
	B cells naive	*R* ═ 0.23, *P* ═ 3.3 e-13	T cells gamma delta	*R* ═ −0.1, *P* ═ 0.0015
	T cells CD4 memory resting	*R* ═ 0.15, *P* ═ 1.3 e-06	Dendritic cells activated	*R* ═ −0.11, *P* ═ 0.00059
	T cells CD4 memory activated	*R* ═ 0.096, *P* ═ 0.0023	B cells memory	*R* ═ −0.11, *P* ═ 0.00043
	Macrophages M1	*R* ═ 0.078, *P* ═ 0.014	Mast cells activated	*R* ═ −0.12, *P* ═ 0.00011
			Macrophages M0	*R* ═ −0.23, *P* ═ 8.5 e-14
		D	Neutrophils	*R* ═ −0.28, *P* < 2.2 e-16
*LIAS*	Mast cells activated	*R* ═ 0.15, *P* ═ 2.7 e-06	T cells CD4 naive	*R* ═ −0.11, *P* ═ 0.00037
	B cells memory	*R* ═ 0.1, *P* ═ 0.00096	Mast cells resting	*R* ═ −0.062, *P* ═ 0.049
	Neutrophils	*R* ═ 0.068, *P* ═ 0.032	T cells CD8	*R* ═ −0.1, *P* ═ 0.0013
			T cells CD4 memory activated	*R* ═ −0.11, *P* ═ 0.00037
			B cells naive	*R* ═ −0.15, P ═ 1.2 e-06
*DLD*	Mast cells resting	*R* ═ 0.078, *P* ═ 0.013	B cells naive	*R* ═ −0.07, *P* ═ 0.028
	Neutrophils	*R* ═ 0.075, *P* ═ 0.018	Macrophages M0	*R* ═ −0.074, *P* ═ 0.02
	Dendritic cells activated	*R* ═ 0.068, *P* ═ 0.032	NK cells resting	*R* ═ −0.11, *P* ═ 5 e-04
*DLST*	T cells CD4 naive	*R* ═ 0.13, *P* ═ 4.7 e-05	Monocytes	*R* ═ −0.087, *P* ═ 0.0061
	B cells naive	*R* ═ 0.12, *P* ═ 0.00016	Neutrophils	*R* ═ −0.14, *P* ═ 9.1 e-06
	NK cells resting	*R* ═ 0.12, *P* ═ 0.00018		
	T cells regulatory (Tregs)	*R* ═ 0.097, *P* ═ 0.0022		
	T cells CD8	*R* ═ 0.071, *P* ═ 0.025		
	T cells CD4 memory activated	*R* ═ 0.069, *P* ═ 0.029		

GLS: Glutaminase.

**Figure 4.**
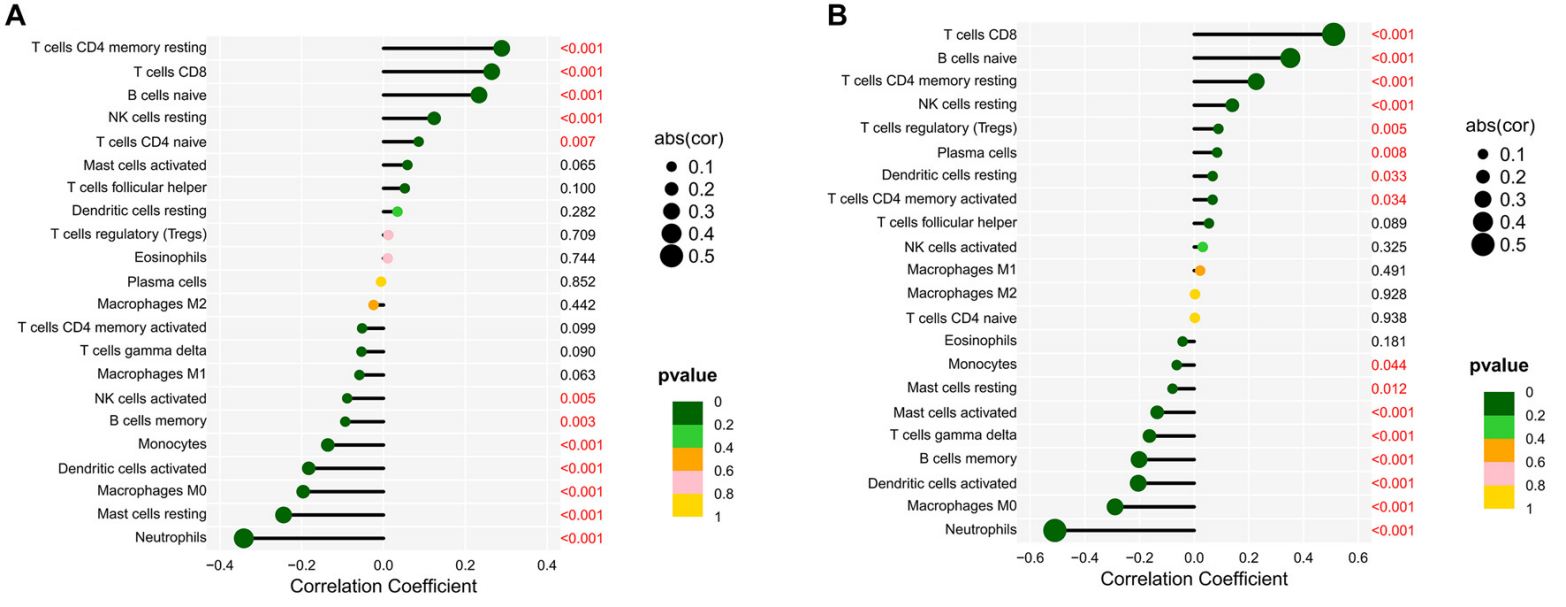
**Lollipop chart of the correlation between two genes and immune cells.** (A) Lollipop chart of *GLS*; (B) Lollipop chart of *PDHB*. GLS: Glutaminase.

As shown in Figure 4A, *PDHB* was significantly and negatively correlated with neutrophils (*P* < 0.001), macrophages M0 (*P* < 0.001), activated dendritic cells (*P* < 0.001), memory B cells (*P* < 0.001), gamma delta T cells (*P* < 0.001), activated mast cells (*P* < 0.001), etc. *PDHB* was significantly and positively correlated with resting NK cells (*P* < 0.001), memory resting T cells CD4 (*P* < 0.001), naive B cells (*P* < 0.001), CD8 T cells (*P* < 0.001), etc.

As shown in Figure 4B, *GLS* was significantly and positively correlated with naive B cells (*P* < 0.001), resting NK cells (*P* < 0.001), memory resting T cells CD4 (*P* < 0.001), CD8 T cells (*P* < 0.001), etc. *GLS* was significantly and negatively correlated with activated dendritic cells (*P* < 0.001), macrophages M0 (*P* < 0.001), resting mast cells (*P* < 0.001), monocytes (*P* < 0.001), neutrophils (*P* < 0.001), etc.

### Analysis of single-cell sequencing data

**Figure 5.**
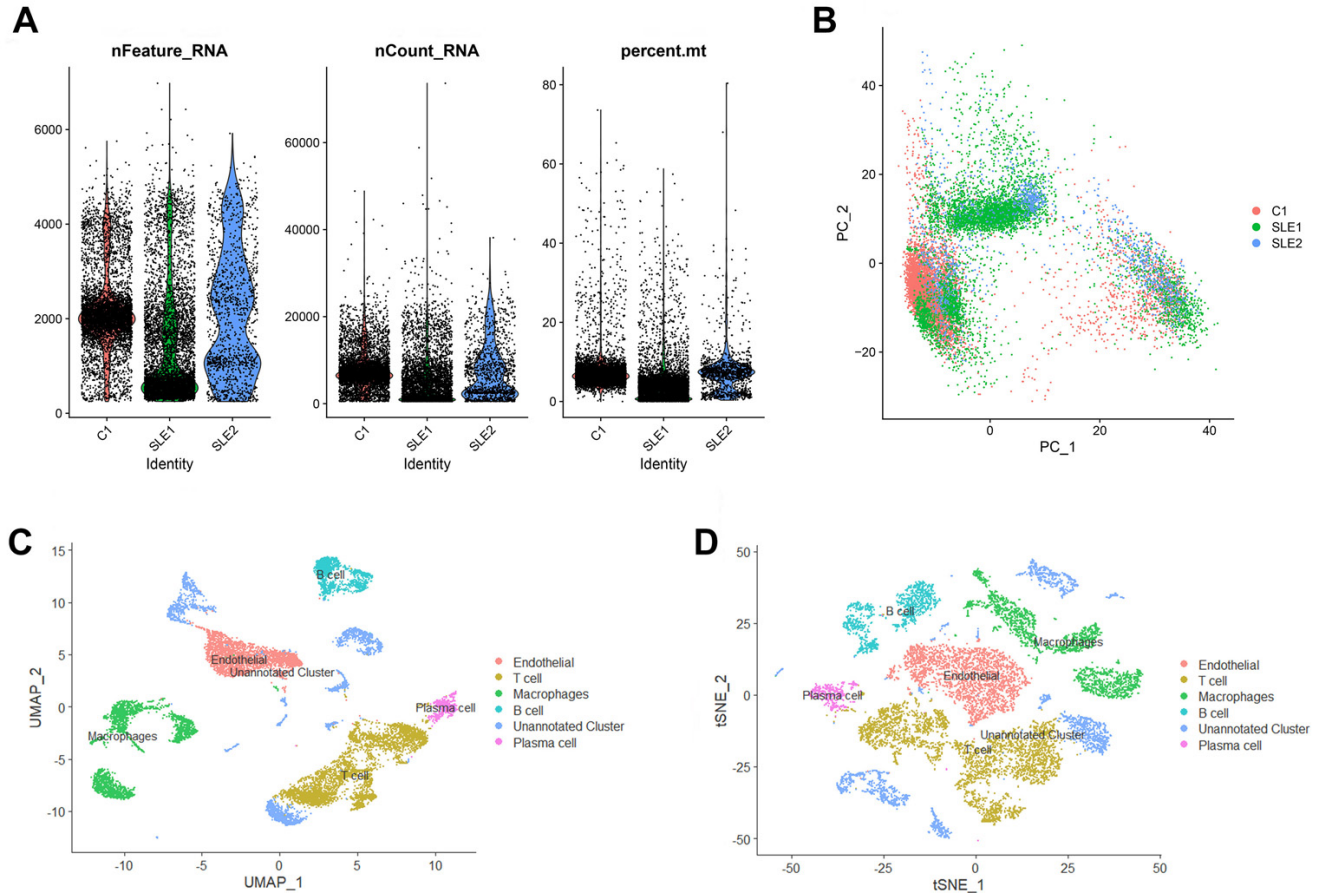
**Analysis of single-cell sequencing data. The single-cell RNA sequencing data were obtained from the GSE162577 dataset in the GEO database.** (A) Violin plot of genetic characteristics of samples; (B) PCA plot colored by various samples; (C) UMAP visualization results after clustering; (D) t-SNE plot colored by various cell clusters. PCA: Principal component analysis; UMAP: Uniform manifold approximation and projection; t-SNE: t-distributed stochastic neighbor embedding.

**Figure 6.**
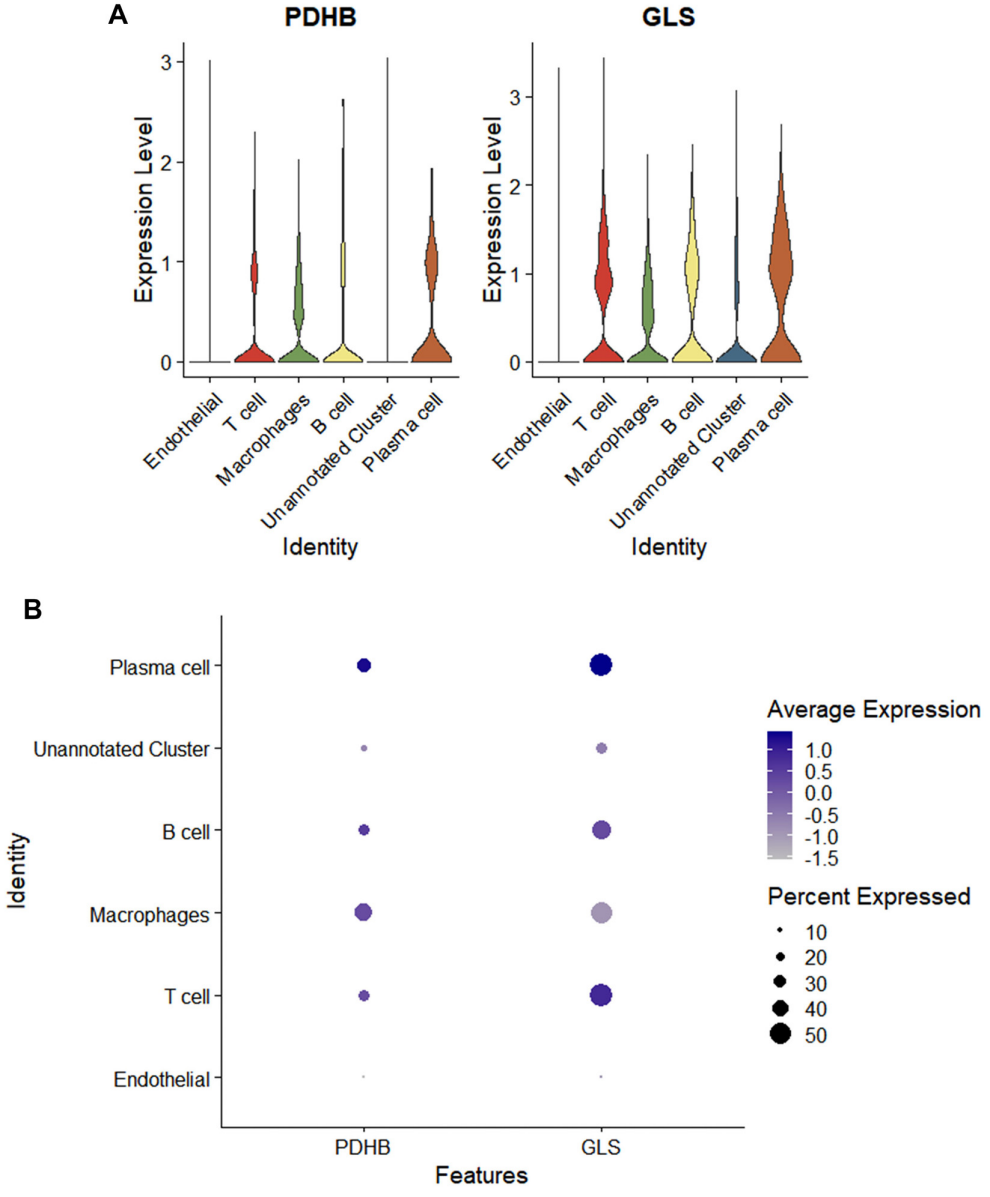
**Distribution of PDHB and GLS in cells**. (A) Violin plot of the distribution of *PDHB* and *GLS* in cells; (B) Bubble plot of the distribution of *PDHB* and *GLS* in cells. GLS: Glutaminase.

We visualized the results to generate violin plots of the genetic signatures from one healthy volunteer and two patients with SLE (Figure 5A). The number of genes in each cell, the number of unique genes in each cell, and the proportion of mitochondria in all cells were obtained (Figure 5A). We used the 10,000 most variable genes (Figure 5B) for dimensionality reduction, which provided PCA results, and used Seurat to identify cell clusters, including endothelial cells, T cells, macrophages, B cells, an unannotated cluster, and plasma cells (Figure 5C). We specifically selected the 10,000 most variable genes based on variance-stabilizing transformation (VST) to ensure the most informative genes were analyzed. This approach allowed us to focus on genes with the highest variability, which are most likely to represent key biological processes relevant to SLE. These selected genes were used for downstream analyses, including dimensionality reduction, clustering, and visualization using PCA, UMAP, and t-SNE. The t-SNE algorithm performed nonlinear dimensionality reduction to produce Figure 5D. *PDHB* showed the highest expression level in plasma cells and the highest proportion in macrophages. *GLS* expression levels and proportions were highest in plasma cells (Figure 6).

### Validation of two significant signature genes

**Figure 7.**
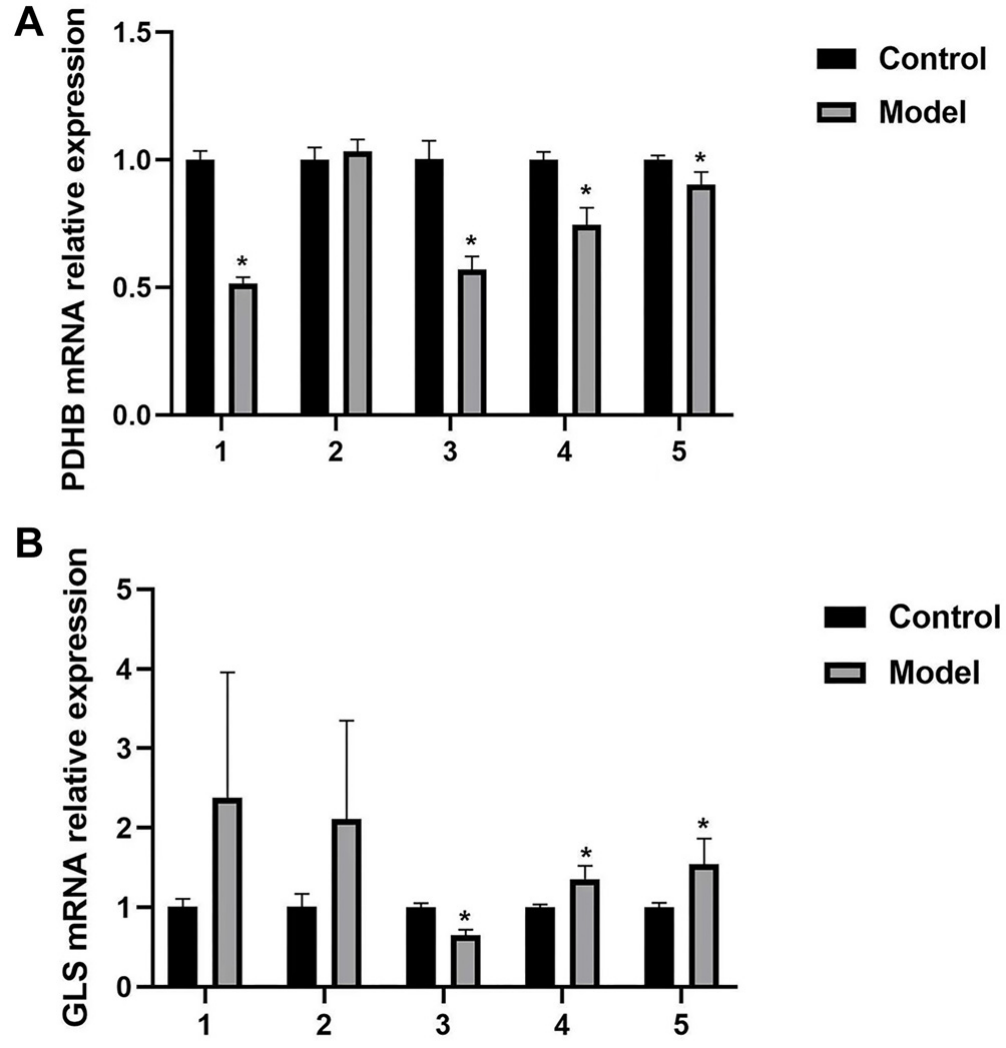
**Validation results of two significant signature genes from MRL/lpr SLE mice and one group of C57BL/6 mice.** (A) Results of *PDHB* expression in treatment and control models; *P < 0.05; (B) Results of *GLS* expression in treatment and control models; *P < 0.05. SLE: Systemic lupus erythematosus; GLS: Glutaminase; lpr: Lymphocyte proliferation.

In this study, MRL/lpr SLE mice were used as the SLE group, and C57BL/6 mice served as the control group. The MRL/lpr SLE mice and C57BL/6 mice were randomly divided into five experimental groups, with six C57BL/6 mice in the control group and six MRL/lpr SLE mice in the SLE group. Both groups of mice were kept under the same conditions for seven days. Afterward, they were euthanized, and peripheral blood was drawn to construct models of the SLE and healthy groups. The mRNA expression of nine diagnostic genes related to cuproptosis in both groups was measured by qPCR. The qPCR results showed that the differences in expression of two key genes, *PDHB* and *GLS*, were statistically significant in all five experimental groups. *PDHB* mRNA expression was significantly low in four of the five MRL/lpr SLE groups (Figure 7A), consistent with the earlier analysis conducted in this study. *GLS* expression was significantly higher in two MRL/lpr SLE groups and lower in one C57BL/6 group (Figure 7B).

## Discussion

We have identified nine signature genes significantly associated with cuproptosis in SLE patients through machine learning screening and validation in animal studies. These genes are *PDHB*, *GLS*, *DLAT*, *FDX1*, *LIPT1*, *MTF1*, *LIAS*, and *DLST*. The results showed that *DLAT*, *DLAT* and *MTF1* were highly expressed in SLE patients, and *LIPT1*, *DLAT*, *PDHB*, *GLS*, *LIAS* and *FDX1* were less expressed in SLE patients than in normal patients.

Through animal experiments, we found that only *PDHB* and *GLS* in mouse experiments were consistent with the machine learning results, while the other genes showed negative results. There have been few studies related to *PDHB* and SLE. One researcher conducted a fine-mapping study of genetic loci and neighboring regions in European SLE samples, revealing an extended region of linkage disequilibrium (LD) (>200 kb) at 3p14.3, which includes the *ABHD6, RPP14, PXK*, and *PDHB* genes, which was extended on *ABHD6, RPP14, PXK,* and *PDHB* genes on 3p14.3.

The final results confirmed the genetic association of locus 3p14.3 with SLE in Europe, pointing to *ABHD6*—but not *PXK*—as the major susceptibility gene in this region [28]. *PDHB* encodes the beta subunit in pyruvate dehydrogenase [28], which catalyzes the first reaction of the oxidative decarboxylation sequence, converting pyruvate to acetyl coenzyme A and CO_2_ [29]. Pyruvate dehydrogenase is a tetramer consisting of two alpha subunits (*PDHA1*) and two beta subunits (*PDHB*). This enzyme, located in mitochondria, is a component of the pyruvate dehydrogenase multienzyme complex (PDH) [29].

*GLS* encodes phosphate-activated glutaminase, the primary enzyme for glutamate production from glutamine [30, 31]. This enzyme may play a key role in behavioral disorders where glutamate acts as a neurotransmitter [32]. *GLS* isozymes, *GLS*1 and *GLS*2, catalyze the first step in glutamine cleavage [33]. *GLS*1 is essential for Th17 cell differentiation, as shown in studies correlating *GLS* with SLE, and modulating *GLS*1 expression improves disease progression in lupus-prone MRL/lpr mice. Inhibiting glutamine catabolism presents a potential therapeutic strategy for SLE by reducing glycolysis and HIF-1α protein expression, thereby affecting metabolic pathways [34]. Some studies have demonstrated that *GLS*2 protein expression is downregulated in CD4+ T cells from lupus-prone MRL/lpr mice and SLE patients. *GLS*2 plays a key role in IL-2 production by CD4+ T cells through antioxidant defense, suggesting that the *GLS*2-inducible pathway is a novel therapeutic target for SLE treatment [35]. Studies on *PDHB* and *GLS* in SLE are limited, and further research is needed.

The other seven genes showed negative results in animal experiments. The *DLST* gene encodes dihydrolipoamide succinyltransferase, a component of the structural core of the alpha-keto glutarate (alpha-KG) dehydrogenase complex in the citric acid cycle (TCA) [36]. *DLST* mutations have been linked to Paragangliomas 7 (PGL7) [37]. *FDX1* encodes Ferredoxin, a small, acidic iron–sulfur protein that acts as an electron transport intermediate for mitochondrial cytochrome P450 and is involved in steroid, vitamin D, and bile acid metabolism [38]. The transfer of lipoic acid to proteins is a two-step process, with the first step involving the activation of lipoic acid by lipoic acid-activating enzymes to form lipoyl-AMP. The second step, transferring the lipoyl group to the apolipoprotein, is carried out by the protein encoded by *LIPT1* [39].

*MTF1*-encoded metallothionein is a small, cysteine-rich protein with a strong affinity for heavy metal ions like zinc, cadmium, and copper and is functionally implicated in heavy metal detoxification and free radical scavenging [40, 41]. *LIAS* encodes lipoic acid synthase. Mutations in this gene have been linked to hyperlipidemia, lactic acidosis, and seizures [42, 43]. The *DLD* gene encodes dihydrolipoamide dehydrogenase. Diseases associated with *DLAT* include dihydrolipoamide dehydrogenase deficiency and maple syrup urine disease [44, 45]. According to the current study, these six genes have not been previously reported in SLE-related articles.

*DLAT* is a protein-coding gene associated with diseases, such as pyruvate dehydrogenase E2 deficiency and liver disease. Among its related pathways are glucose/energy metabolism and pyruvate metabolism [46]. It has been suggested that mitochondrial phagocytic processes may limit the secretion of inflammatory factors and directly regulate mitochondrial antigen presentation and immune cell homeostasis. Autophagy induction has also been associated with enhanced expression of *DLAT* on the cell surface [47]. After validation, the association of these genes with SLE still requires further discussion.

Through machine learning combined with gene expression profiling, we identified genes associated with cuproptosis and validated some of them. This offers new insights into the pathogenesis and diagnosis of SLE, as well as potential targeted therapies, though further investigation is required for their clinical application.

## Conclusion

In conclusion, our findings provide valuable insights into the role of cuproptosis in SLE and highlight the potential diagnostic value of *PDHB* and *GLS* in this disease.

## Data Availability

The data that support the findings of this study are available in Gene Expression Omnibus (datasets with identifiers: GSE65391, GSE61635, and GSE162577), which were downloaded from http://www.ncbi.nlm.nih.gov/geo/.
